# Force Sensor for Instrumented Patellar Prostheses: Development and Characterization

**DOI:** 10.3390/s25041226

**Published:** 2025-02-18

**Authors:** Vera Maioli, Matteo Zauli, Angelo Cappello, Luca Cristofolini

**Affiliations:** 1 Department of Industrial Engineering, School of Engineering and Architecture, Alma Mater Studiorum, University of Bologna, Via Umberto Terracini 24-28, 40131 Bologna, Italy; vera.maioli2@unibo.it; 2 CIRI ICT, Alma Mater Studiorum, University of Bologna, Viale Risorgimento 2, 40136 Bologna, Italy; matteo.zauli7@unibo.it; 3 Department of Electrical, Electronic and Information Engineering, Alma Mater Studiorum, University of Bologna, Viale Risorgimento 2, 40136 Bologna, Italy

**Keywords:** piezoresistive, force sensor, instrumented prosthesis, patella, patellofemoral joint, total knee arthroplasty

## Abstract

The development of an instrumented patellar prosthesis, able to measure the contact forces at the patellofemoral joint, can significantly aid in investigating the causes of total knee arthroplasty failures due to patellar complications. This study focuses on developing and validating an instrumented patellar prosthesis to measure contact forces in the patellofemoral joint. A piezoresistive force sensor was characterized and integrated into a conditioning circuit, with the aim of its implementation in the prosthesis. To measure medial and lateral forces independently, the sensors were trimmed in half. Compression tests (up to 2000 N) assessed sensor performance in terms of linearity (R^2^ = 0.998 intact vs. 0.989 trimmed), repeatability (0.9% intact vs. 0.8% trimmed), and accuracy (1.7% intact vs. 2.3% trimmed) for forces up to 250 N. Higher force levels resulted in increased errors, but at a rate still comparable to that of existing sensors in the literature. Key considerations for the design of the instrumented prosthesis, such as minimizing point and shear loads, were identified. A prototype prosthesis capable of housing the sensor was proposed. The integrated system shows potential for improving the understanding of Total knee arthroplasty (TKA) failures through in vitro studies and could serve as an intraoperative tool for the evaluation of bone resections.

## 1. Introduction

Total knee arthroplasty (TKA) is a generally successful and cost-effective treatment for alleviating pain and improving functionality in patients with arthritis [1]. However, patient satisfaction remains low, with only approximately 20% of patients reporting overall satisfaction after surgery [2]. Additionally, 75% of patients do not achieve a natural joint [3], and 50% experience residual symptoms [4]. Among these symptoms, patellar complications are particularly common; Rand et al. [5] reported a 12% revision rate for TKA due to complications related to the extensor mechanism. These complications include issues such as patellar fractures, patellar tendon ruptures, recurring patellar subluxations, patellar pain, malrotated patella, wear, loosening, the rupture of the patellar implant, patellar necrosis, and fracture.

The prosthesis alters joint kinematics, consequently creating abnormal contact forces between the femur and the patella [6]. As a result, numerous studies have focused on investigating the kinematics and contact forces of the patellofemoral (PF) joint. 

Several studies utilized smart sensors to detect contact forces in the PF joint. Most of these studies used pressure-sensitive color-changing film sensors, such as Prescale Fuji [7,8], or resistive film pressure sensors, such as the Scan I and Scan K by Tekscan (e.g., [9,10]). These sensors offer the advantage of being relatively thin and enable the collection of data related to pressure and the contact area between two surfaces. While color-changing tactile sensors are limited to recording the pressure peak at each spot, Tekscan sensors can capture real-time data. However, all these sensing techniques exhibit relatively poor accuracy, providing a more qualitative rather than quantitative pressure distribution assessment. In fact, most of the studies published where Tekscan sensors are used provide limited detail about measurement errors. One of the few studies where the errors of the Tekscan Scan I and Scan K were assessed reported a root mean squared error of 6.5% ± 4.4% for the resultant force measurement, and of 0.86% ± 0.58% for force distribution measurement, when seven levels of force corresponding to mean pressures of 1–7 MPa were applied [11].

Additionally, despite their thin profile, both sensors struggle to conform to the intricate double curvature of the knee joint. These sensors are provided in fixed sizes which sometimes are larger than necessary. When adopted to measure pressure inside the knee joint, this excess material can interfere with surrounding structures like ligaments and the joint capsule, ultimately disrupting natural kinematics. Moreover, both sensors are applied at the interface of the joint, between the femoral trochlea and the patella. Therefore, the sensors are curved to fit the geometry of the articular surface. The stiffness of these sensors leads to incomplete adhesion in the double curvature of the joint, thus leading to inevitable measurement error. In addition, the sensors were subjected not only to axial loads, but also to shear, which could compromise the measurements, if not even ruin them.

To avoid these problems, sensors inserted inside the prosthesis itself, rather than at the joint interface, have already been used in other types of prostheses in the past. This guarantees that the sensors work under optimal conditions in terms of geometry and interfaces. Prosthetic sensing has already been used extensively, originally in the hip stem [12], and later in the knee [13] and shoulder [14]. Telemetric prostheses with semiconductor strain gages [15] have been used to measure the in vivo loads during daily activities. Piezoelectric pressure sensors have been used to measure intraoperatively the forces delivered to prosthetic components. A feasibility study of a knee prosthesis which incorporated piezoresistive sensors has been published [16].

Piezoresistive sensors are devices utilized for detecting mechanical quantities and converting them into electrical signals. They operate based on the physical principle of piezoresistance, involving the variation in the electrical resistivity of a material when subjected to mechanical load [17]. Piezoresistive sensors are particularly advantageous for measuring pressures in prosthetic implants due to their thin profile and suitability for high-frequency dynamic measurements. To the best of the authors’ knowledge, they have never been used in patellar prosthesis, and an assessment of their degree of uncertainty is lacking in the literature, especially under off-label loading conditions.

Implementing such an instrumented patella could be very effective during the in vitro testing phase of new prosthetic designs. In particular, measuring separately the forces transferred from the medial and from the lateral trochlea to the patellar component would provide extremely valuable insights into load distribution and joint stability. This could also support the development of an intraoperative device to verify the correct resection of the patellar bone before implantation. Having a measure of the mismatch of forces acting in the medial and lateral parts of the patella may help to prevent prosthesis malpositioning. 

In this study, we propose the use of piezoresistive sensors inside a patellar prosthesis, an approach that has never been used in the literature, particularly in relation to assessing forces in both the medial and lateral compartments of the knee. 

The objective of this study was to lay the foundation for developing an instrumented patellar prosthesis capable of measuring forces at the PF joint both in the medial and in the lateral compartment. Specifically, we aimed to achieve the following:

1. Build a circuit capable of reading changes in sensor resistance and transmitting them as a voltage signal to a computer for reading, processing, and analysis;
2. Characterize the sensor using the constructed circuit, evaluating its repeatability, linearity, and accuracy under a variety of loading and boundary conditions;
3. Evaluate potential performance losses due to cutting the sensor, which is necessary for the proper placement of the medial and lateral sensors within the prosthesis;
4. Explore the sensor’s performance under suboptimal loading conditions, an aspect that has not been frequently addressed in previous studies. The tests performed with localized loads, shear forces, and misalignment are especially valuable for understanding the sensor’s robustness and reliability in scenarios different from ideal conditions, which may occur during daily in vitro testing, or during possible intraoperative use;
5. Identify the key points that must be addressed for proper sensor operation and design an instrumented patellar prosthesis incorporating the sensor while meeting the identified specifications.

## 2. Materials and Methods

### 2.1. Requirements

The successful integration of a force sensor into a patellar prosthesis requires careful consideration of the technical specifications of the sensor and its electronic circuitry. These components must meet specific functional and environmental criteria to guarantee reliable performance under biomechanical conditions. The force sensor in a patellar prosthesis is subjected to dynamic and repetitive loads. Although it is not intended for in vivo applications, normal activities such as walking, running, and climbing stairs can be reproduced during in vitro testing. Consequently, the sensors must be capable of accurately measuring contact forces both under static conditions and during complex simulated motions. The following specifications are necessary for the sensor to function effectively:

- Thickness: To be implemented inside a patellar prosthesis, the sensor must be sufficiently thin. Considering the typical thickness of commercial patellar components, which ranges from less than 3 mm in the outermost part to 10 mm in the dome, a maximum thickness of 0.5 mm was chosen for the sensor.
- Diameter: The diameter of commercial patellar prostheses varies between 25 and 40 mm, differentiated into a range of sizes. The smallest sizes in the market are seldom used in Western countries, as the average diameter of a patella is around 35–55 mm. As a consequence, the position of the resultant force can vary within a large area. If the resultant force has an offset (i.e., is not centered with the sensor), this results in a combination of a force and a bending moment, which translates to an uneven load distribution. For the purpose of this project, to reduce the consequences of a force offset, the sensor must cover the load-bearing area of the prosthesis to ensure accurate force measurement in the regions subjected to the highest loads during typical activities. To achieve this, the sensor diameter is smaller than 35 mm overall (to fit within the prosthesis) but features a sensitive area larger than 25 mm to provide comprehensive coverage of the load-bearing region and improve measurement quality.
- Shape: in order to obtain two separate measurements of the force transmitted through the medial and lateral components, the sensors must have a semicircular shape or otherwise be able to be adapted to the desired shape.
- Measurement Range: Studies conducted in vivo have measured reaction forces at the patellar joint that can reach up to 5000 N during running and 900 N during walking [18]. Loads during the in vitro or intraoperative application of the sensor are not likely to exceed such a range. Indeed, during in vitro tests, the forces applied at the quadriceps tendon typically fall in the range of 175–600 N [19]. For this range of quadriceps forces, we expect to measure a maximum patellar contact force in the range of 250–900 N. In intraoperative use, given the absence of quadriceps contraction, we expect even lower forces. However, in order to expand the possible use of the instrumented prosthesis, the range is extended to 0–2000 N.
- Measurement Accuracy: The signal from the force sensor should be as insensitive as possible to an offset of the applied force, and to the presence of force components other than the one perpendicular to the prosthesis itself. Possible strategies for minimizing the shear force components delivered to the sensor through prosthetic design will be discussed later (Section 4.1).
- Cost: At this stage, the sensor was designed for in vitro use, as it is possible that the sensor or its connectors are broken during implantation, testing, or removal. Therefore, we aimed for a single-use low-cost solution. This is also in line with possible future intraoperative use, where a single-use solution would overcome all the issues related to (re)sterilization.
- Power consumption: no special conditions of durability or power consumption are required.

### 2.2. Architecture

To meet the requirements listed above, we chose the FlexiForce A401 Tekscan (Tekscan Inc., Boston, MA, USA). This is a thin (0.203 mm), flexible, pressure-sensitive sensor constructed from a flexible printed circuit board. Its operation principle relies on piezoresistivity: the resistance of the sensors depends on the force applied in a rather linear fashion. It has a total diameter of 31.8 mm, with a diameter of the sensitive area of 25.4 mm. These sensors can withstand forces up to 30 kN without damage. The useful range can be set by adjusting the gain of the conditioning circuit. 

**Figure 1:**
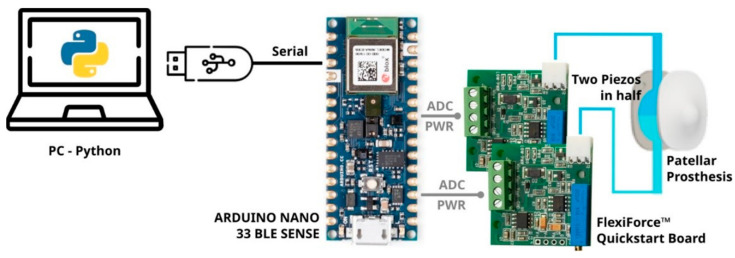
Acquisition system from the instrumented patella implant through the conditioning units.

In order to measure forces acting in the medial and lateral compartments of the PF joint separately, it was necessary to trim the sensors in half. Each trimmed sensor had to be conditioned and acquired separately (Figure 1). The conditioning circuit used was the FlexiForce™ Quickstart Board by Tekscan, enabling changes in resistance to be transformed into voltage measurements. The development board was the Arduino Nano 33 BLE Sense (Arduino, Ivrea, Italy). This uses the nRF52840 microcontroller unit (MCU) and is equipped with an analog-to-digital converter. The measurements taken were then transmitted via USB to a computer for processing using a Python script, enabling real-time data collection. 

Two trimmed piezoresistive sensors were connected to this battery-powered system to provide continuous measurement during the tests. This circuit utilizes an inverting operational amplifier arrangement to produce an analog output based on sensor resistance. The gain of the conditioning circuit can be adjusted with a potentiometer as needed. The maximum signal that can be fed to the MCU utilized is 3.3 V. Therefore, the potentiometer of the conditioning circuit was set so that an output of 3.3 V corresponded to a force of 2200 N applied to the sensor. The output values were processed with a Python code developed to convert the signal output into force values and compared with the testing machine used to deliver the loads (see Section 2.3).

### 2.3. Characterization of the Sensor System

After verifying that the sensitivity and linearity characteristics of the sensor were not compromised by the signal transmission circuit, tests were carried out on four intact specimens of the chosen sensor and on the same specimens after being trimmed in half. All the results reported refer to the data collected downstream of the entire circuit. 

Tests were performed using a uniaxial servo-hydraulic testing machine (Instron 8500 controller, Instron, UK) equipped with a 10 kN load cell and a custom loading setup. After a series of preliminary tests, to avoid the errors deriving from a force distributed over an excessive area, it was decided to deliver the force to the sensor by applying a puck. This is a cylinder (0.7 mm high) made of Delrin^®^ Acetal Plastic, which allows for a reduction in the contact area to 70% of the active sensitive area of the sensor, as suggested by the manufacturer.

Each sensor was conditioned before characterization by applying a load equivalent to 110% of the maximum test load (2200 N) for 5 s 3 times. Conditioning was repeated after trimming, before the characterization of the trimmed sensors. To avoid temperature effects, the room temperature was kept within a range of 21–27 °C. The specimens were tested as follows:

- To assess linearity over the range, 13 levels of force between 0 and 2000 N were applied, with each level being repeated three times. The applied force values were not evenly distributed between 0 and 2000 N but were concentrated at lower values (0–250 N), as this range is more relevant to the practical application of the sensor;
- To further evaluate the repeatability of the sensor at a specific load level in the middle of the range, five repetitions at a nominal force of 1000 N were performed.

The following error indicators were computed:

- Linearity was evaluated considering the coefficient of determination (R2) of the linear regression between the imposed and the output force values. The same assessment was repeated both over the entire range (0–2000 N) and over the reduced force range (0–250 N);
- The repeatability error was calculated as the ratio between the standard deviation and the mean of the measured outputs at the same imposed load, at each of the load levels described above;
- The accuracy error was calculated as the difference between the imposed force (as measured by the load cell of the testing machine) and the readout from the sensor. The root mean squared error (RMSE) was computed and reported as a percentage of the readout.

The prosthesis will be modified (Section 4.1) so as to deliver force to the sensor over the largest possible area, as close as possible to the sensor’s center, and with as small as possible shear forces. However, the actual application of force to the sensor cannot be expected to correspond to ideal conditions. For these reasons, to test the sensitivity of the sensor under suboptimal loading conditions, the following additional tests were conducted:

- To evaluate the effect of a force distributed over an area smaller than the intended one, the tests were repeated so that the force was delivered to the sensor through a spherical tip. To test this configuration, three load repetitions at 250 N and 500 N were applied to the sensor. Higher forces were not applied in these tests to avoid damaging the sensor;
- To evaluate the effect of a force delivered with an offset (i.e., not exactly in the center of the sensor), measurements were taken by centering the force with respect to the sensor, and also by creating an offset of 8 mm (i.e., 2/3 of the radius of the sensor);
- To evaluate the effect of residual shear forces, the tests were repeated with the specimens tilted by 18° (this value depended on the loading rigs available in the lab).

## 3. Results

Measurements were successfully performed for all the loading conditions. Both from the tests on intact specimens and those after trimming in half, it was clear that each sensor needed to be calibrated separately as the gain varied between specimens by 76%.

**Figure 2:**
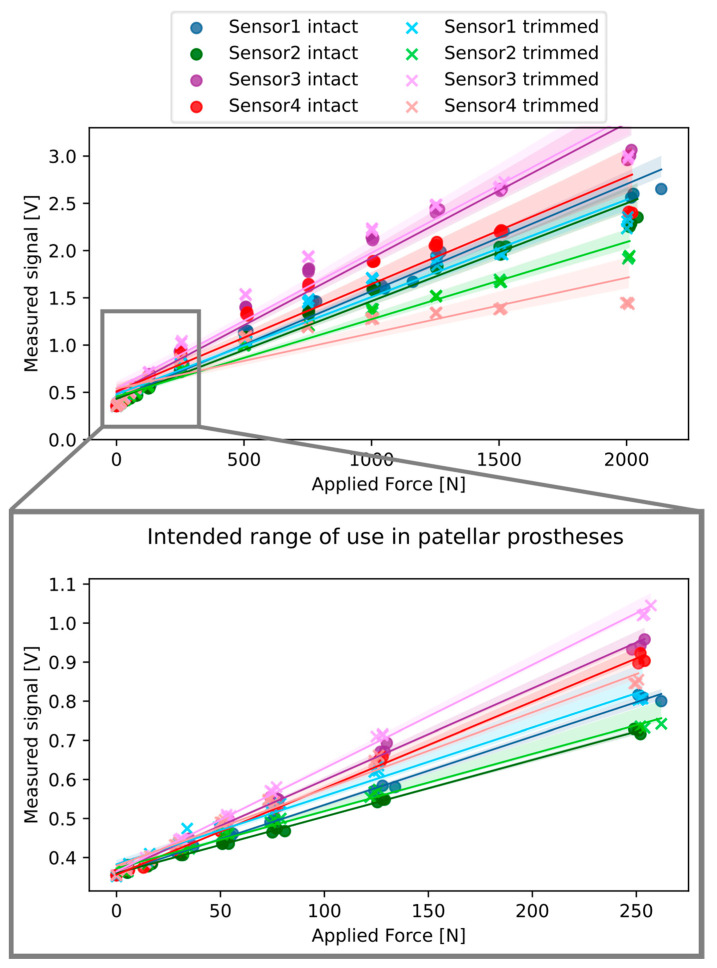
Signal outputs of the four specimens before and after being trimmed in half. Both the entire range of forces tested (top) and the intended range of use inside the patellar prosthesis (zoomed below) are shown. The lines represent the linear regression for each sensor, while the transparent area represents the confidence interval.

All specimens showed a nonlinear trend, with a slightly decreasing slope as the applied force was increased, although the maximum signal output of 3.3 V was never reached (Figure 2). The curves showed better linearity over a smaller range of forces (Table 1). Moreover, the differences in performance between the intact and trimmed specimens were smaller in a smaller range of forces. The maximum difference calculated in the same specimen between the intact and trimmed condition was 58% over the entire range (0–2000 N) and 12% over the reduced range (0–250 N).

The maximum standard deviation among the four specimens across five repetitions at a force of 1000 N was 39.8 N for the intact sensor and 39.6 N for the trimmed sensor (Table 1). 

All specimens had a repeatability better than 1.9% at 1000 N. Across the full measurement range (0–2000 N), repeatability was consistently better than 1.3% for all specimens. While some trimmed sensors showed slightly improved repeatability compared to their intact counterparts (e.g., sensors 1 and 4 at 1000 N), this variation did not appear to be systematic. This parameter was not altered by trimming.

Accuracy was higher when the specimens were tested over the smaller force range (0–250 N) compared to the full range (0–2000 N) (Table 1). After trimming, accuracy generally declined for all specimens (except sensor 3), particularly at higher force levels (Table 1).

**Figure 3:**
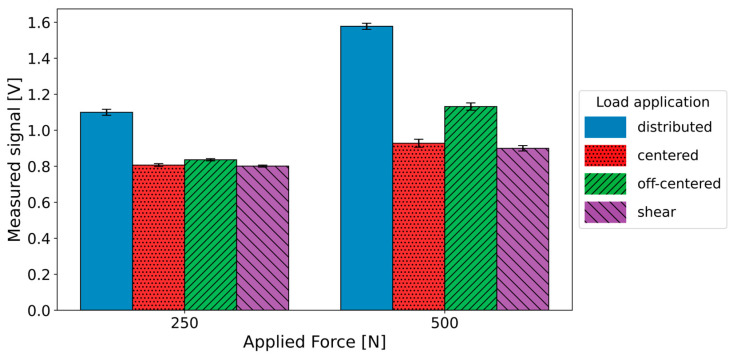
Signal outputs with distributed and concentrated centered and off-centered force, and loads with shear components when a force of 250 N (on the left) and 500 N (on the right) is applied. The average and standard deviation over three repetitions are plotted.

The results with centered and off-centered concentrated loads as well as loads with shear components preserved a repeatability always better than 1.5% (Figure 3). However, when the applied force was concentrated over a smaller area, the sensor underestimated the actual force by 26.7% and 41.2% for 250 N and 500 N, respectively. When the force was applied with an offset of 8 mm, the sensor underestimated the actual force by 23.9% (250 N) and 28.3% (500 N). When the force included a shear component, the sensor underestimated the perpendicular force component by 27.1% (250 N) and 43.0% (500 N).

## 4. Discussion

The aim of this study was to develop an instrumented patellar prosthesis capable of detecting contact forces in the medial and lateral components of the PF joint. This included characterizing the force sensor and deriving guidelines for its correct usage in an instrumented patellar prosthesis. 

Based on the specifications for the application of a force sensor inside a patella prosthesis, the FlexiForce A401 piezoresistive sensor was chosen, and the signal transmission circuit was built (Figure 1). We verified that the signal transmission circuit did not alter the sensor characteristics, and we tested the linearity, repeatability, and accuracy of four specimens of the chosen sensor. The specimens were then cut in half, and the same tests were repeated to guarantee that there was no loss of performance (Table 1). 

A decrease in linearity was observed in the trimmed sensors, especially when considering applied force up to 2000 N. Limiting the range to 250 N reduced or eliminated this loss of linearity. However, the results showed excellent repeatability, with no detrimental effects of trimming. Similar observations were made regarding accuracy. For a force range up to 250 N, accuracy was excellent (RMSE 1.7% ± 0.4% for intact and 2.3% ± 0.7% for trimmed specimens). These results can be compared with those of a study where resistive pressure films were used, where an accuracy of 6.5% ± 4.4% was reported for a smaller measurement range [11]. 

All the specimens tested showed some nonlinearity: the slope slightly decreased as the applied force increased (Figure 2). This nonlinearity should not be a problem in our particular application, since the forces during in vitro testing are expected to be less than 250 N; in this range, the specimens showed excellent linearity (Table 1). 

Each sensor displayed a unique calibration curve, and trimming the sensor altered this curve (Figure 2). Therefore, it was fundamental to perform calibration on each individual trimmed sensor to guarantee accurate measurements. Once the specimens were calibrated, the linearity error was 3.2% ± 2.4% for forces up to 250 N. Repeatability errors were always smaller than 2%. These parameters indicate that the sensors are highly reliable for this application, namely, for the range of forces of the intended application. When the sensors were tested with forces up to 2000 N, the repeatability error remained at 2%, but the linearity error increased to 13.2% ± 5.5%. For this range of forces, it may be necessary to adopt nonlinear regression. While forces up to 2000 N are not expected in our specific application, this information may be relevant for other applications, such as in vitro tests with higher quadriceps forces. Moreover, to our knowledge, there is a lack of calibration curves for Tekscan Force A401 sensors in the literature, so we believe it is important to provide this additional information for broader use.

However, it is important to note that these results were obtained with axial force distributed over a sufficiently large sensor area. In the suboptimal scenario where the force was concentrated over a small area, a sharp reduction in sensor gain was noted, but not in repeatability (Figure 3). 

As reported by the manufacturer, the piezoresistive material has some nonlinearity. Therefore, the same force distributed over different areas translates to different output signals. Furthermore, the manufacturer indicates that the pressure delivered to the Tekscan FlexiForce A401 should not exceed 70 MPa. When the load is uniformly distributed across the sensor’s surface, the pressure remains safely below this threshold. However, with concentrated loads, either at the center or on the periphery, the local pressure could exceed the threshold, which significantly reduces the sensor’s sensitivity and leads to an underestimation of the applied force. It is important to keep this limitation in mind when designing the instrumented patellar prosthesis and deciding where to insert the sensor. For practical applications, it is therefore critical to design the contact areas so as to distribute the applied forces. It is also important to avoid the application of shear forces, which can damage the sensor and affect measurement accuracy (Figure 3). 

### 4.1. Further Development of the Instrumented Prosthesis

Given the results obtained and the observations derived, the patellar prosthesis was successfully modified to host the sensor while meeting the following requirements:

- The modified patellar component should provide flat support for the sensor;
- The internal surfaces of the modified patellar component should guarantee that the forces are as evenly distributed as possible over an area that is at least 70% of the sensitive area of the sensor (as recommended by the manufacturer);
- The puck machined inside the prosthesis should minimize the impact of forces acting outside the sensitive area of the sensor;
- The connection between the prosthetic components and the sensor should minimize shear forces by using low-friction materials and/or a design that will minimize shear force components transferred across the sensor;
- Unlike several solutions already published [20,21], the external geometry of the prosthesis and the method of implantation should not be modified, so as not to alter the kinematics of the PF joint.

**Figure 4:**
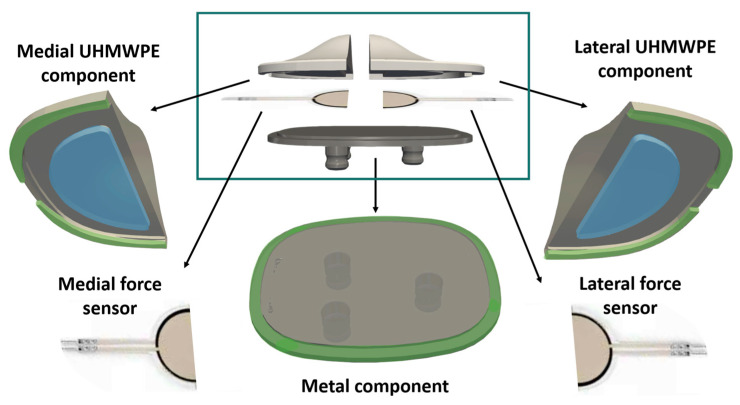
Customized patellar prosthesis with the application of two force sensors. In the top center square is an exploded view of the assembly; on the sides are its components: the medial component made of UHMWPE, the lateral component made of UHMWPE, two TekScan Flexiforce A401 force sensors trimmed in half, and the metal component at the base. The pucks are highlighted in blue and transfer the entire compressive force. The connection edges (in green) are designed so as to provide only an in-plane constraint without transferring any compressive force.

A commercial resurfacing patella (GMK resurfacing patella, Medacta International S.A., Castel San Pietro, Switzerland) was customized to accommodate the sensors while preserving the very same external dimensions and shape. The instrumented patella (Figure 4) consisted of three parts:

- The first was a part that fitted into the patellar bone, made of a 3D-printed Ti6Al4V titanium alloy, which was modified internally to provide a flat base for the sensor;
- The patella also included a part that articulated into the femur, made of UHMWPE, which was divided in turn into a medial and a lateral part. Each of the two halves was provided with a semicircular puck, with a diameter equal to 70% of the diameter of the sensitive area of the sensor. These pucks were necessary to adequately distribute the forces over the desired sensor area and mitigate the effects of shear forces. The UHMWPE components were provided with edges connecting the metal and polymeric parts of the patellar prosthesis so as to ensure that shear forces were transmitted through these edges (rather than transferred to the sensors). The sensors were strategically positioned to maximize surface area coverage and were aligned with the geometric dome of the implant. The pucks of each UHMWPE component were centered on the corresponding sensor. Moreover, the arrangement of the two sensors was independent, enabling the separate measurement of the medial and lateral forces.

To prevent the prosthesis from disassembling, these components were held together not only by the interlocking edges but also by the adhesive layer of the sensor.

### 4.2. Study Limitations and Future Work

While the instrumented patellar prosthesis met all design requirements and demonstrated promising results during initial testing, certain limitations should be acknowledged. First, the mechanical behavior of the modified prosthesis was only tested under controlled laboratory conditions. Further in vitro testing under more realistic loading conditions, such as cyclic loading and joint motion, is required to confirm its durability and reliability. Second, the adhesive layer used to hold the components together may be susceptible to wear or detachment over time, particularly under extreme loading or temperature variations. Future work will explore alternative fixation methods to ensure stability and reliability for the entire duration of possibly long test sessions. Finally, while the current design supports the independent measurement of medial and lateral forces, additional sensors could be incorporated in future iterations to assess shear force distribution more precisely.

Future developments could also include implementing a wireless data transmission system to improve the usability of the instrumented prosthesis during in vivo testing or clinical applications.

## 5. Conclusions

The aim of this study was to develop an instrumented patellar prosthesis to measure the forces transmitted to the femoral component. The piezoresistive force sensors selected presented excellent linearity and repeatability within the useful force range for ex vivo or intraoperative applications within the patellofemoral joint. The accuracy was significantly better than that of the most widely used sensors in the field. Careful consideration of the load distribution and avoidance of shear forces is essential, as is the need to calibrate each sensor after trimming prior to implantation. Finally, a patellar component design capable of containing two force sensors was proposed to measure the medial and lateral components of contact forces at the patellofemoral joint. 

## Figures and Tables

**Table 1:** Measurements of linearity, repeatability, and accuracy for the four specimens tested, before and after trimming in half.

	Sensor1	Sensor2	Sensor3	Sensor4	Average
**Intact sensors**					
R^2^ (0–2000 N)	0.986	0.978	0.969	0.939	0.968
R^2^ (0–250 N)	0.997	0.998	0.998	0.998	0.998
Standard Deviation [N] (1000 N)	37.65	37.95	38.95	39.83	-
Repeatability Error at 1000 N	1.85%	1.47%	0.59%	1.15%	1.26%
Mean Repeatability Error (0–2000 N)	1.04%	1.16%	0.88%	0.66%	0.93%
Accuracy RMSE (0–2000 N)	3.9%	5.0%	5.7%	8.4%	5.7%
Accuracy RMSE (0–250 N)	2.1%	2.0%	1.8%	1.0%	1.7%
**Trimmed sensors**					
R^2^ (0–2000 N)	0.964	0.972	0.951	0.852	0.935
R^2^ (0–250 N)	0.983	0.991	0.998	0.986	0.989
Standard Deviation [N] (1000 N)	39.36	39.31	39.05	39.56	-
Repeatability Error (1000 N)	0.47%	1.42%	0.51%	0.45%	0.71%
Mean Repeatability Error (0–2000 N)	1.29%	0.72%	0.71%	0.63%	0.83%
Accuracy RMSE (0–2000 N)	6.3%	5.6%	7.4%	12.9%	8.0%
Accuracy RMSE (0–250 N)	3.0%	2.3%	1.1%	2.8%	2.3%

## Data Availability

The datasets used and/or analyzed during the current study are available from the corresponding author upon reasonable request.

## Abbreviations

The following abbreviations are used in this manuscript:

TKA	total knee arthroplasty
PF	patellofemoral
MCU	microcontroller unit
RMSE	root mean squared error
